# The Type II Hsp40 Sis1 Cooperates with Hsp70 and the E3 Ligase Ubr1 to Promote Degradation of Terminally Misfolded Cytosolic Protein

**DOI:** 10.1371/journal.pone.0052099

**Published:** 2013-01-16

**Authors:** Daniel W. Summers, Katie J. Wolfe, Hong Yu Ren, Douglas M. Cyr

**Affiliations:** Department of Cell and Developmental Biology, School of Medicine, University of North Carolina at Chapel Hill, Chapel Hill, North Carolina, United States of America; University of Pittsburgh, United States of America

## Abstract

Mechanisms for cooperation between the cytosolic Hsp70 system and the ubiquitin proteasome system during protein triage are not clear. Herein, we identify new mechanisms for selection of misfolded cytosolic proteins for degradation via defining functional interactions between specific cytosolic Hsp70/Hsp40 pairs and quality control ubiquitin ligases. These studies revolved around the use of *S. cerevisiae* to elucidate the degradation pathway of a terminally misfolded reporter protein, short-lived GFP (slGFP). The Type I Hsp40 Ydj1 acts with Hsp70 to suppress slGFP aggregation. In contrast, the Type II Hsp40 Sis1 is required for proteasomal degradation of slGFP. Sis1 and Hsp70 operate sequentially with the quality control E3 ubiquitin ligase Ubr1 to target slGFP for degradation. Compromise of Sis1 or Ubr1 function leads slGFP to accumulate in a Triton X-100-soluble state with slGFP degradation intermediates being concentrated into perinuclear and peripheral puncta. Interestingly, when Sis1 activity is low the slGFP that is concentrated into puncta can be liberated from puncta and subsequently degraded. Conversely, in the absence of Ubr1, slGFP and the puncta that contain slGFP are relatively stable. Ubr1 mediates proteasomal degradation of slGFP that is released from cytosolic protein handling centers. Pathways for proteasomal degradation of misfolded cytosolic proteins involve functional interplay between Type II Hsp40/Hsp70 chaperone pairs, PQC E3 ligases, and storage depots for misfolded proteins.

## Introduction

Cells are routinely challenged by changes in growth conditions that perturb protein homeostasis. The action of protein quality control (PQC) machinery is essential to maintain levels of non-native proteins within a tolerable range [1]. Inefficiencies in PQC result in the accumulation of misfolded polypeptides as amorphous aggregates, toxic oligomers, and amyloid-like species, all of which threaten cellular homeostasis. In the cytosol there is constant flux of non-native proteins through the Hsp70 system with the life or death of chaperone clients being determined by specialized co-chaperones [2], [3], [4]. The Hsp70 network manages non-native clients through multiple strategies including promotion of refolding, suppression of aggregation, and facilitation of degradation [3], [5]. Hsp70 also acts to facilitate the assembly of ordered amyloid-like aggregates that serve as a sink for aberrant protein conformers and thereby sequester toxic protein species [6], [7]. The Hsp70/Hsp40 system may also facilitate the entrance and exit of non-native clients to one of three different misfolded protein handling centers; 1) the IPOD which is located at the cell periphery adjacent to the vacuolar membrane and contains amyloid-like, detergent insoluble aggregates 2) the JUNQ which is perinuclear and contains detergent soluble aggregates 3) a peripheral compartment that is enriched in the small heat shock protein Hsp42 and contains detergent soluble aggregates [6], [8], [9], [10]. However, the rules that determine whether a non-native polypeptide is concentrated to one compartment versus another are unknown and the fate of proteins packaged into these assemblies is not clear.

Hsp40s represent a large family of Hsp70 co-chaperones that are essential regulators of the ATP hydrolytic cycle of Hsp70 and target Hsp70 to specialized machineries and cellular locations [11], [12], [13]. The Hsp40 family is subdivided into three classes (Types I–III) with all members containing a J-domain that interacts with Hsp70 and additional specialized domains that mediate substrate binding and/or target Hsp70 to different quality control machines [13], [14], [15]. The most abundant Hsp40s are members of the Type I and Type II sub-families who partner with Hsp70 to promote protein folding [12], [16], protein degradation [17], [18], [19], [20], translation [21], translocation across membranes [22] and assembly of amyloid-like fibers [23]. In eukaryotes such as yeast, the Type I and Type Hsp40s Ydj1 and Sis1 utilize their unique structural features, substrate specificity, post-translational modification, and localization to direct Hsp70 to function in different aspects of protein metabolism [10], [24], [25], [26], [27], [28]. Yet, it is still unclear how specialized Hsp70:Hsp40 pairs function in PQC networks to triage non-native clients for folding, degradation, or sequestration into misfolded protein handling centers.

To define Hsp70-dependent steps in triage decisions that lead to protein degradation in the eukaryotic cytosol we expressed in yeast a terminally misfolded and short-lived chimeric GFP fusion protein (slGFP). SlGFP has an N-terminal domain that is too short to fold into a stable conformation fused to tandem GFPs, so its fate can be monitored visually and biochemically. Therefore, study of slGFP degradation provides a valuable approach to define chaperone dependent steps in protein degradation without having to consider interpretations related to folding.

We report that Hsp70 cooperates with Sis1 and the PQC E3 ligase Ubr1 to mediate proteasomal degradation of slGFP. Interestingly, attenuation of Sis1 or Ubr1 activity lead slGFP to accumulate in a Triton X-100-soluble state and be packaged into protein handling centers that are visualized as cytosolic puncta. The slGFP that accumulated in puncta when Sis1 activity was low was subsequently degraded in a proteasome dependent manner. Yet, in the absence of Ubr1, puncta localized slGFP was relatively stable. The Sis1/Hsp70 system and Ubr1 cooperate in degradation of a terminally misfolded cytosolic protein. Cells compensate for saturation of the Sis1/Ubr1 E3 machinery via storage of degradation competent protein assemblies in cytosolic puncta. Terminally misfolded proteins that accumulate in PQC puncta can subsequently be degraded in a process that requires Ubr1.

## Materials and Methods

### Yeast Strains, Growth Conditions, And Reagents

Yeast strains and plasmids are listed in Tables S1 and S2 respectively. Strains were transformed using the lithium acetate transformation method. Yeast were grown to mid-log phase under selection to maintain plasmids and incubated at 30°C throughout each experiment. Cells transformed with pESC-GFP-VHL were grown overnight in 2% galactose as previously described [18]. Bortezomib (Sigma) was dissolved in DMSO as a 100 mM stock immediately prior use. Anti-GFP antiserum was purchased from Roche. Anti-Hsp104 antiserum was from Stressgen. Antisera to Ydj1, Sis1 and Ssa1 were used as previously described [10], [29]. Anti-PGK1 was from Molecular Probes. Anti-Flag (M2) was from Sigma.

### Cycloheximide-Chase Analysis Of SlGFP Degradation

Yeast cultures expressing the indicated proteins were treated as described in the text. To inhibit protein translation, cultures were treated with 200 µg/mL cycloheximide and aliquots were removed at indicated times. Cells were lysed by alkaline pretreatment [30]; cells were pelleted from culture media and resuspended in 0.1 M NaOH and incubated for 5 min at room temperature, washed in sterile H_2_O, and boiled for 15 min in denaturing lysis buffer (60 mM Tris-HCl pH 6.8, 2%SDS, 2 mM DTT). Cell lysates were precleared at 3,000 rpm for 3 min and the protein concentration of supernatants were normalized. Normalized lysates were diluted in sample buffer (60 mM Tris-HCl pH 6.8, 2% SDS, 10% glycerol, 2 mM EDTA, 5% β-mercaptoethanol, 1 mg/mL bromophenol blue) then analyzed by SDS-PAGE and western immunoblotting for the indicated proteins.

### Co-Immunoprecipitation Of Complexes Between SlGFP And Chaperones Proteins

Yeast strains expressing the indicated proteins were lysed by glass bead disruption in Buffer A (150 mM NaCl, 50 mM Hepes pH 7.4, 1 mM EDTA, 0.1% Triton X-100, 1 mM PMSF, and 1× yeast protease inhibitor cocktail [Roche]). Cell extracts were precleared at 3,000× G for 3 min at 4°C. The supernatant was saved and protein concentrations assessed with a BioRad protein determination kit. Protein concentrations were normalized between samples to approximately 3 mg/mL and 300 µg of protein was incubated with the indicated antisera for 1 hour at 4°C then incubated with Protein G beads (50% slurry preblocked with BSA) for 30 min at 4°C. Beads were washed 2–3 times with Buffer A then resuspended in sample buffer and analyzed by SDS-PAGE and immunoblotting.

### Detection Of Ubiquitinated SlGFP

Yeast strains expressing slGFP were grown under selection to mid-log phase. Cells were washed with cold H_2_O (+1 mM NaN_3_ and 20 mM NEM) and lysed by glass bead disruption in Buffer A (+1 mM NEM). Cell extracts were precleared at 3,000 rpm for 3 min at 4°C, protein concentrations normalized, and GFP immunoprecipitated with anti-YFP antisera and protein G resin using standard methods. Protein G resin was washed three times in Buffer A supplemented with 0.1% SDS. Ubiquitinated slGFP was detected after SDS-PAGE and western immunoblotting of precipitated material for ubiquitin (Covance). Levels of ubiquitinated slGFP were quantified using laser densitometry and ImageJ software (NIH) and normalized as a ratio to the level of slGFP that was immunoprecipitated from the lysate (detected using anti-GFP).

### Fluorescence Microscopy of SlGFP

Yeast strains expressing the indicated proteins and treated as described in the text were fixed in 3.7% formaldehyde and stored in phosphate buffered saline (pH 7.5) supplemented with 1.2 M sorbitol. Fixed cells were permeabilized and DNA visualized with DAPI as described in [10]. Rnq1-mRFP was expressed from the *GAL1* promoter for 4 hours before cells were processed for analysis. Cells were visualized with an Olympus IX81 Fluorescence microscope and images processed with Metamorph software. Exposure times and all other settings were standardized across individual experiments unless otherwise noted.

### Centrifugation Of Cell Extracts For Determination Of SlGFP Solubility In Triton X-100

Cells were lysed by glass bead disruption in Buffer A (+1 mM DTT) and lysates pre-cleared at 3,000 rpm for 3 min at 4°C. The supernatant was saved and a quantity of lysate that contained 200 µg of protein was spun at 100,000× G for 30 min at 4°C. An aliquot was saved prior to the spin to represent the total input. Equivalent volumes from total, supernatant, and pellet fractions were added to 2× sample buffer and analyzed by SDS-PAGE and western immunoblotting for the indicated proteins.

## Results

### SlGFP Is A Substrate Of Hsp70 And Hsp40 And Degraded By The Proteasome

**Figure 1:**
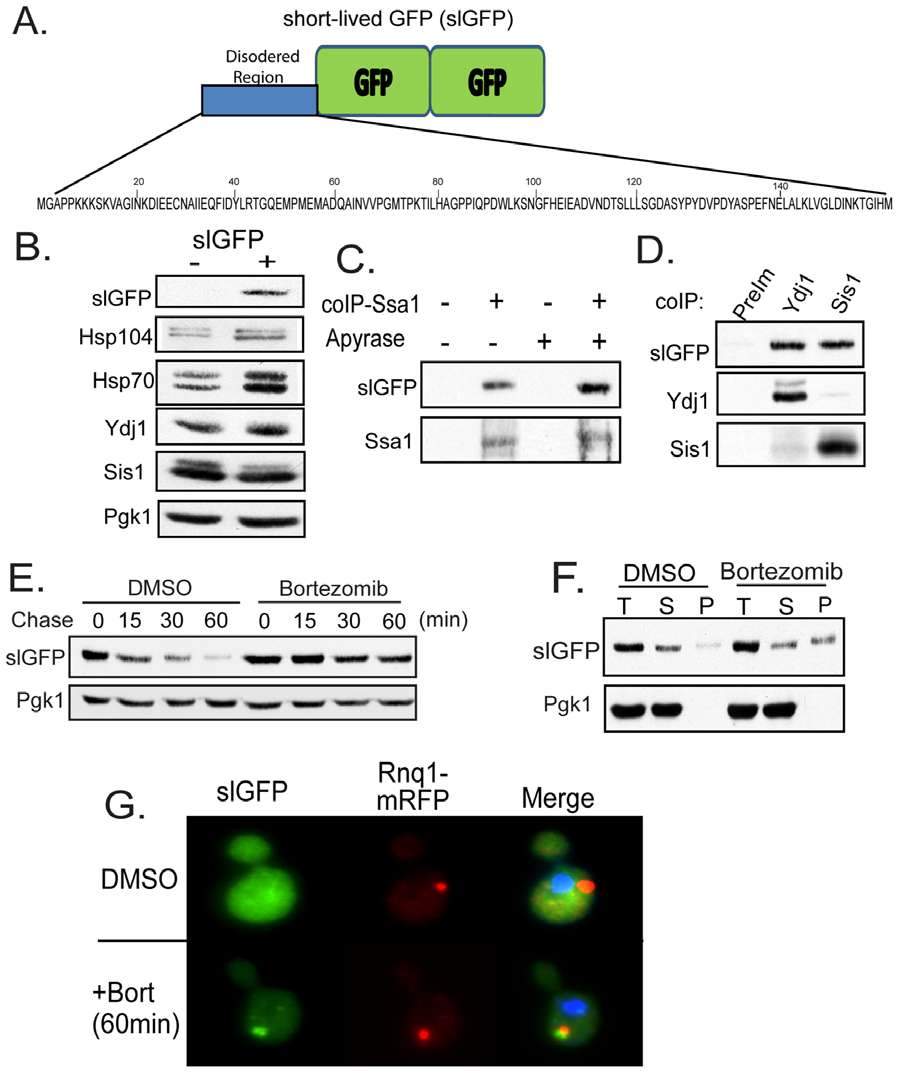
SlGFP Is Degraded By The Ubiquitin-Proteasome System. A) Domain structure of slGFP containing an N-terminal, unstructured region and two GFP moieties. The sequence of the N-terminal domain is shown below the schematic. B) Changes in chaperone levels in a strain expressing slGFP. C) SlGFP co-immunoprecipitated with Hsp70 Ssa1 in cell extracts treated with or without Apyrase. D) SlGFP co-immunoprecipitated with the Hsp40s Sis1 and Ydj1. E) Cycloheximide chase analysis of slGFP levels in the presence or absence of the proteasome inhibitor bortezomib. Cells were pretreated with DMSO or 100 µM bortezomib for 15 min then slGFP turnover was monitored by western blot at indicated chase times. F) Triton X-100 solubility of slGFP after a 60 min bortezomib treatment. G) Fluorescence microscopy of cells expressing slGFP and Rnq1mRFP after a 60 min bortezomib treatment.

To analyze how protein degradation and aggregation pathways intersect in the cytosol we examined the degradation pathway of a labile and aggregation-prone cytosolic protein. This protein (herein referred to as slGFP for short-lived GFP) consists of a 126 amino acid N-terminal domain containing several unstructured regions that are enriched in hydrophobic motifs and putative Hsp70/Hsp40 chaperone binding sites (Fig. 1A and Fig. S1A) [27], [31], [32]. SlGFP was originally designed to shuttle in and out of the nucleus as it contains nuclear localization and export signals (NLS and NES) [33], but these motifs have no impact on the degradation kinetics of slGFP (Fig. S1B). SlGFP is subject to both nuclear and cytoplasmic quality control and dual GFP moieties at its C-terminus allow visualization of its intracellular fate by fluorescence microscopy. Consequently, SlGFP serves as an important tool to study cytosolic chaperone function in disposal of misfolded or damaged multi-domain proteins.

To maximize detection of slGFP's flux though PQC pathways, slGFP was expressed from the constitutive *ADH1* promoter. Importantly, slGFP expression from this promoter did not impact cell growth although we observed elevation in levels of heat-shock inducible chaperones Ssa1 and Hsp104. However, slGFP did not induce a global heat shock response since the levels of Ydj1 and Sis1 were unchanged (Fig. 1B). SlGFP is indeed a substrate for cytosolic chaperones as it co-immunoprecipitated with Hsp70 Ssa1 (Fig. 1C). This interaction appeared specific as the presence of slGFP in precipitates with Hsp70 was enhanced by depletion of ATP from cell lysates with Apyrase. SlGFP could also be co-immunoprecipitated with the cytosolic Hsp40s Ydj1 and Sis1 (Fig. 1D).

In addition to being a chaperone substrate, slGFP has a short half-life of 15 min and its degradation is blocked by inhibition of the proteasome (Fig. 1E). At steady-state, slGFP exists in a Triton X-100-soluble state (Fig. 1G). However, under conditions of proteasome inhibition a large fraction of slGFP accumulated in a Triton X-100-insoluble state (Fig. 1F).

We were curious about the fate of slGFP that accumulated upon proteasome inhibition and examined its behavior by fluorescence microscopy. Under normal growth conditions, slGFP is predominantly diffuse in the cytosol and its visualization required long exposure times reflecting the unstable nature of this protein (Fig. 1G). Yet, proteasome inhibition resulted in slGFP redistribution into a single punctate structure that co-localizes with the IPOD marker Rnq1-mRFP (Fig. 1G) [8]. Proteasome inhibition also induced slGFP relocalization in the absence of Rnq1-mRFP expression (not shown). These observations collectively demonstrate that slGFP is an Hsp70 chaperone substrate that is degraded by the proteasome and inhibition of the proteasome results in slGFP accumulation in the IPOD. Therefore, slGFP behaves like a canonical misfolded cytosolic protein and is a useful tool for the study of how cytosolic PQC factors partition terminally misfolded proteins between pathways for degradation and aggregate packaging.

### SlGFP Degradation Requires The Quality Control E3 Ubiquitin Ligases Ubr1 And San1

**Figure 2:**
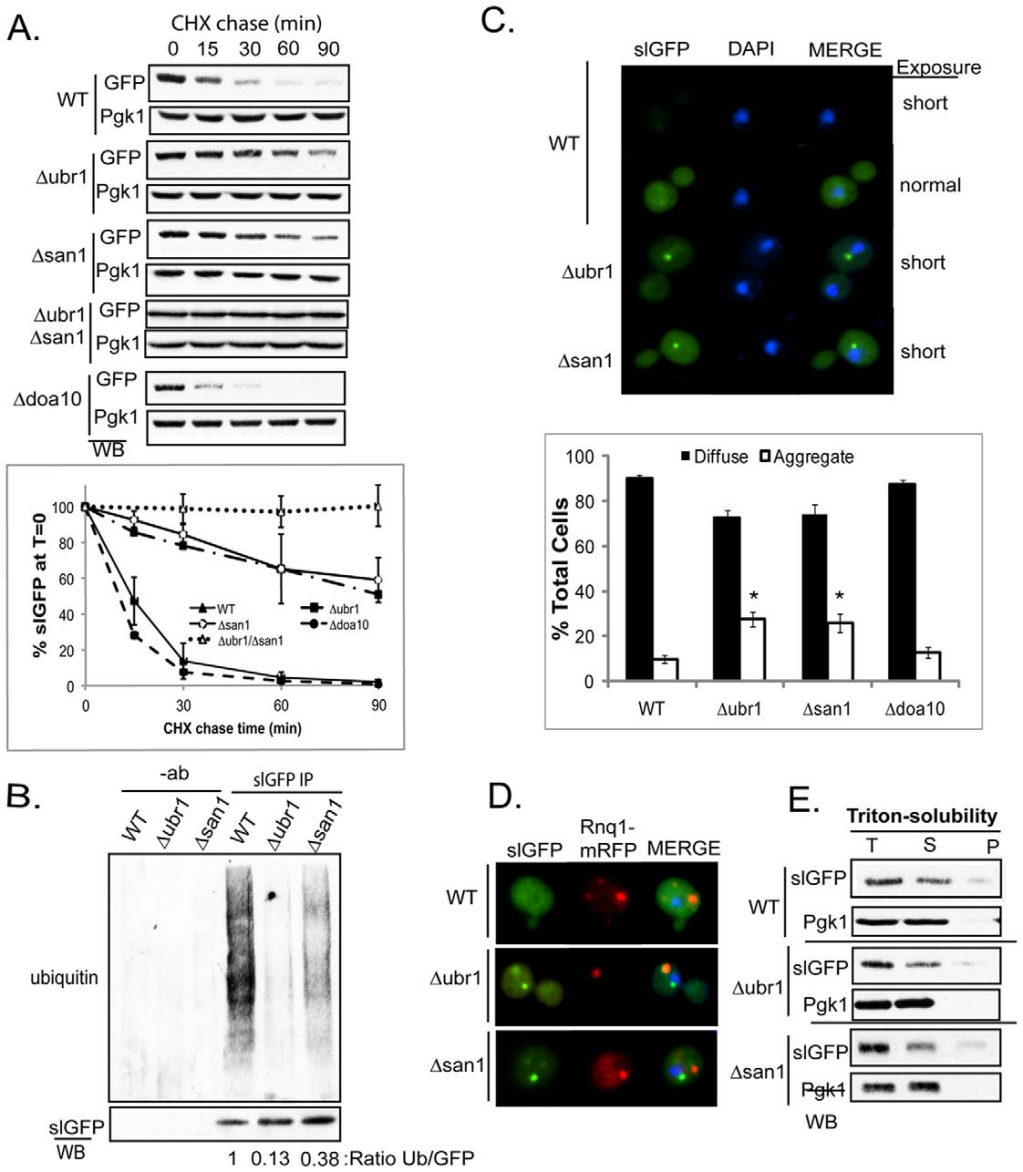
Ubr1 And San1 E3 Ligases Participate In Degradation Of SlGFP. A) Turnover of slGFP in WT, *Δubr1*, *Δsan1* or *Δdoa10* was assessed by cycloheximide chase analysis. Quantification of three independent experiments is shown below. B) Ubiquitination of slGFP in WT, *Δubr1*, or *Δsan1*. SlGFP was immunoprecipitated from cell extracts and immunoprecipitated material was analyzed by western immunoblotting for ubiquitin. C) Localization of slGFP was analyzed in WT, *Δubr1*, or *Δsan1* by fluorescence microscopy. SlGFP fluorescence was notably lower in a WT strain background so a longer exposure was required to compare slGFP localization with *Δubr1* or *Δsan1*. Quantification of slGFP puncta in indicated strain backgrounds is shown on the right (*p<0.05 n = 500 cells in three independent experiments). Error bars represent ± SEM. D) Co-localization of slGFP puncta in WT, *Δubr1*, or *Δsan1* strains with Rnq1-mRFP by fluorescence microscopy. DAPI is blue. E) Detergent solubility of slGFP in WT, *Δubr1*, or *Δsan1* was assessed after high-speed centrifugation (T-total, S-supernatant, P-pellet).

The E3 ubiquitin ligases Ubr1, San1, and Doa10 participate in PQC and mediate selection of misfolded cytosolic proteins for degradation [17], [34], [35], [36], [37], [38]. We wondered what role each might play in the pathway for slGFP degradation. The half-life of slGFP was greatly extended in *Δubr1* and *Δsan1*, but not in *Δdoa10* (Fig. 2A). In *Δubr1 Δsan1* degradation of slGFP was not detected during the time course of a cycloheximide chase (Fig. 2A). Decreased rates of slGFP degradation correlated with dramatic reductions in steady-state levels of ubiquitinated slGFP in *Δubr1* and *Δsan1* (Fig. 2B). Since we do not detect ubiquitinated slGFP in *Δubr1*, we could not assay for further a reduction in slGFP ubiquitination in Δubr1 Δsan1. Overall, these data suggest that slGFP is degraded via PQC networks that utilize the E3s Ubr1 or San1, but not Doa10.

Since slGFP forms Triton X-100-insoluble aggregates and relocalizes to the IPOD upon proteasome inhibition, we investigated how reducing slGFP ubiquitination would affect its solubility and localization. Interestingly, in ***Δ*** *ubr1* or *Δsan1* strains, slGFP formed cytosolic puncta in a significantly higher percentage of cells (Fig. 2C). Yet, in contrast to what was observed upon proteasome inhibition, slGFP puncta formed in E3 deletion strains were often localized proximal to the nucleus (Fig. 2B), and did not co-localize with the IPOD marker Rnq1-mRFP (Fig. 2D). In addition, aggregates that accumulated in *Δubr1* or *Δsan1* were soluble in Triton-X100 (Fig. 2E). Triton-X100 soluble proteins are detected in both the JUNQ and peripheral compartment and markers for these assemblies overlap [6], [8], [9], [10]. Thus, we simply refer to the foci that contain the Triton-X100 soluble forms of slGFP as puncta. Accumulation of slGFP in puncta in ***Δ*** *ubr1* or *Δsan1* was observed when the carbon source for growth was glucose (Fig. 2B) or galactose (Fig. 2C) and occurred independent of Rnq1 expression (Fig. 2B). Thus, slGFP is degraded rapidly in a manner that is perturbed by inactivation of a cytosolic or nuclear PQC E3 and impairment of slGFP ubiquitination results in its accumulation in puncta known to contain Triton X-100 soluble proteins.

### Elevating Sis1 Promotes SlGFP Degradation

**Figure 3:**
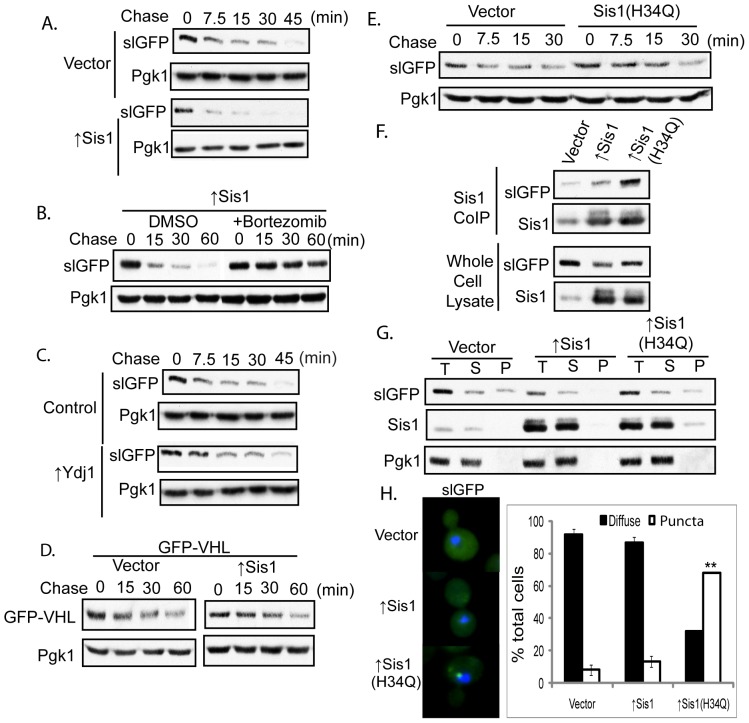
Elevating Sis1 Accelerates SlGFP Degradation. A) Cycloheximide chase analysis of slGFP turnover in cells transformed with an empty vector or Sis1 overexpression plasmid. B) Cells overexpressing Sis1 were pre-treated with DMSO or 100 µM bortezomib for 15 minutes and slGFP turnover analyzed by cycloheximide-chase analysis. C) SlGFP turnover in cells expressing Ydj1. D) Turnover of GFP-VHL. E) SlGFP turnover in cells expressing Sis1(H34Q) or an empty vector. F) Cells overexpressing Sis1 or Sis1(H34Q) were lysed and Sis1 was co-immunoprecipitated from cell extracts (top panel). The lower panel shows slGFP and Sis1 levels from whole cell extracts. G) Triton X-100-solubility of slGFP in cells expressing Sis1 or Sis1(H34Q), T-total, S-supernatant, P-pellet. H) SlGFP localization in cells expressing Sis1 or Sis1(H34Q). Quantification of cells with slGFP aggregates is shown below (**p<0.01). Error bars represent ± SEM from three independent experiments and a total of 500 cells were analyzed for each condition.

The behavior of slGFP upon PQC E3 inactivation suggests that subtle perturbation of the cell's capacity to ubiquitinate and degrade a misfolded protein may result in the accumulation of this protein in puncta. As the Hsp40 chaperones Ydj1 and Sis1 interact with slGFP, we investigated whether altering the levels of these chaperones impacts PQC of this unstable protein. Elevating Sis1 accelerated slGFP degradation, decreasing its half-life from 15 min to approximately 5 min (Fig. 3A). Sis1-mediated slGFP degradation is proteasome-dependent because slGFP was stabilized in the presence of elevated Sis1 by treatment of cells with the proteasome inhibitor bortezomib (Fig. 3B). In contrast, overexpressing Ydj1 had little effect on the rate of slGFP turnover (Fig. 3C).

A specific role for a Type II Hsp40 such as Sis1 in the degradation of a misfolded protein has not been demonstrated, so we evaluated whether elevating Sis1 levels had the same effect on a different non-native protein. While slGFP is terminally misfolded, other non-native proteins exist in equilibrium between native and non-native states. For example, folding of von Hippel Lindau (VHL) protein requires partner proteins (elongin B & C) and molecular chaperones to fold properly [39], [40], [41]. Upon expression of VHL in yeast, in the absence of these partner proteins, VHL is degraded, but the half-life of VHL is longer than we observe for slGFP [18]. Overexpression of Sis1 had no effect on VHL turnover kinetics, which suggests that Sis1 selectively promotes degradation of some, but not all misfolded cytosolic proteins (Fig. 3D).

We expected that Sis1 was acting through its partner Hsp70 Ssa1 to accelerate slGFP turnover. To determine if Sis1 action in slGFP degradation is indeed Hsp70-dependent, a mutation was made in the Sis1 J-domain (H34Q) that disrupts a conserved Hsp70 binding motif [15]. Overexpression of Sis1(H34Q) did not accelerate slGFP degradation, rather we observed an increase in the slGFP half-life (Fig. 3E). Under conditions where expression levels of Sis1 and Sis1(H34Q) were equivalent, Sis1(H34Q) binding to slGFP was strongly enhanced (Fig. 3F); this result would be expected if Sis1(H34Q) were no longer able to transfer the slGFP it binds to Hsp70. The slGFP that accumulated upon overexpression of Sis1(H34Q) remained soluble in Triton X-100 (Fig. 3G), but a significant increase in the percentage of cells with slGFP puncta was detected (Fig. 3H). Collectively, these data suggest that Sis1 binds slGFP and cooperates with Hsp70 to promote slGFP degradation. Compromise of Sis1 function results in a pool of detergent soluble slGFP degradation intermediates being concentrated into cytosolic puncta.

### Reduction Of Sis1 Levels Hinders SlGFP Degradation

**Figure 4:**
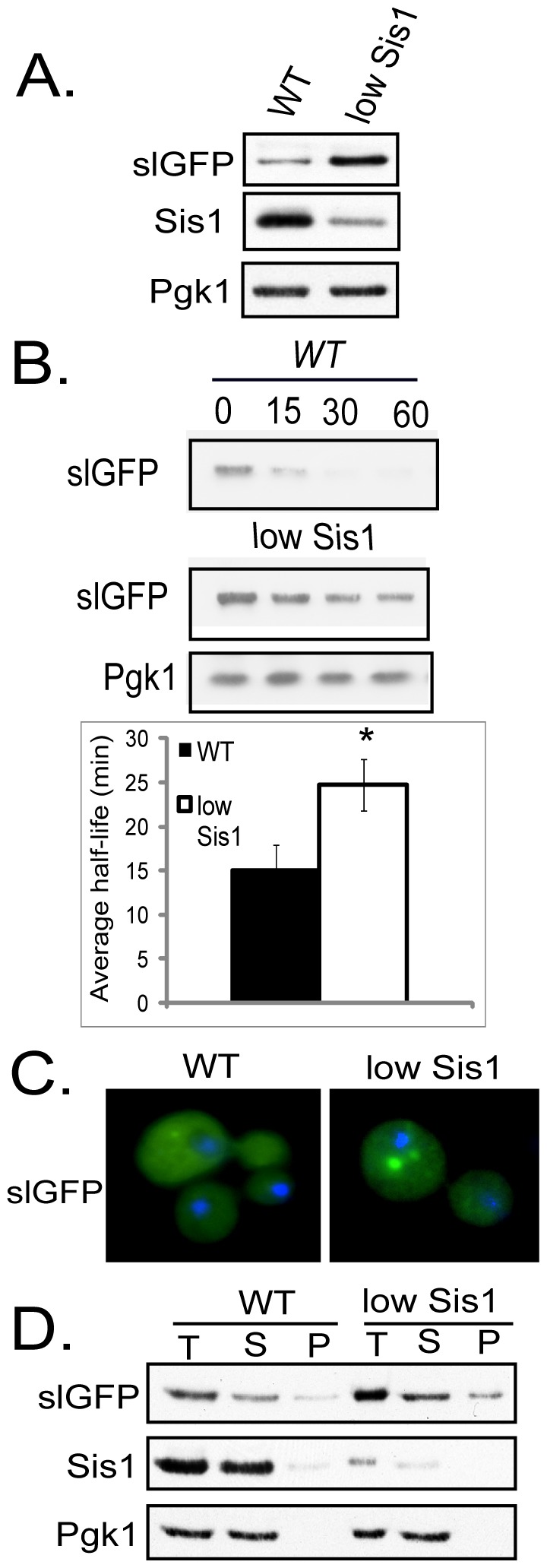
Reducing Sis1 Levels Stabilizes SlGFP and Induces SlGFP Accumulation in Puncta. A) Steady-state slGFP levels in an isogenic WT strain and *Δsis1* complemented with *SIS1* expressed from a plasmid under control of the doxycycline-repressible promoter (low Sis1). B) Cycloheximide-chase analysis of slGFP turnover in indicated strains. Quantification of the half-life of slGFP in a WT or low Sis1 strain. Error bars represent the SEM for three independent experiments. C) Fluorescence microscopy of slGFP localization. D) Triton X-100-solubility of slGFP.

Sis1 appears to be a critical player in slGFP degradation and studies with Sis1(H34Q) suggest that blocking Sis1 function impacts localization of slGFP in the same manner as inactivation of Ubr1 or San1. To explore this further, we evaluated the impact of reducing Sis1 levels on the solubility and degradation of slGFP. As *SIS1* is essential we investigated slGFP degradation in *Δsis1* complemented with a plasmid in which *SIS1* is expressed under control of tetracycline repressible promoter. Sis1 levels in this strain were 85% lower than in an isogenic wild type strain even in the absence of doxycycline, though growth rates were unaffected (Fig. 4A, and data not shown). This strain was termed low Sis1 and use of it afforded the opportunity to monitor slGFP degradation under conditions where Sis1 levels were low, but cell viability was unchanged. In the low Sis1 strain, accumulation of slGFP was elevated and the half-life of slGFP was increased almost 2-fold (Fig. 4A and 4B). Further reducing Sis1 to undetectable levels by treatment of the low Sis1 strain with doxycycline further extended slGFP half-life (Fig. S2).

In the low Sis1 strain there was also a striking increase in the number of cells that contained slGFP foci (Fig. 4C). Similar to a *Δubr1* or *Δsan1* strain, the slGFP in low Sis1 remained predominantly Triton X-100-soluble (Fig. 4D). When levels of Sis1 are low, slGFP degradation is hindered and slGFP degradation intermediates appear to accumulate in detergent-soluble puncta.

**Figure 5:**
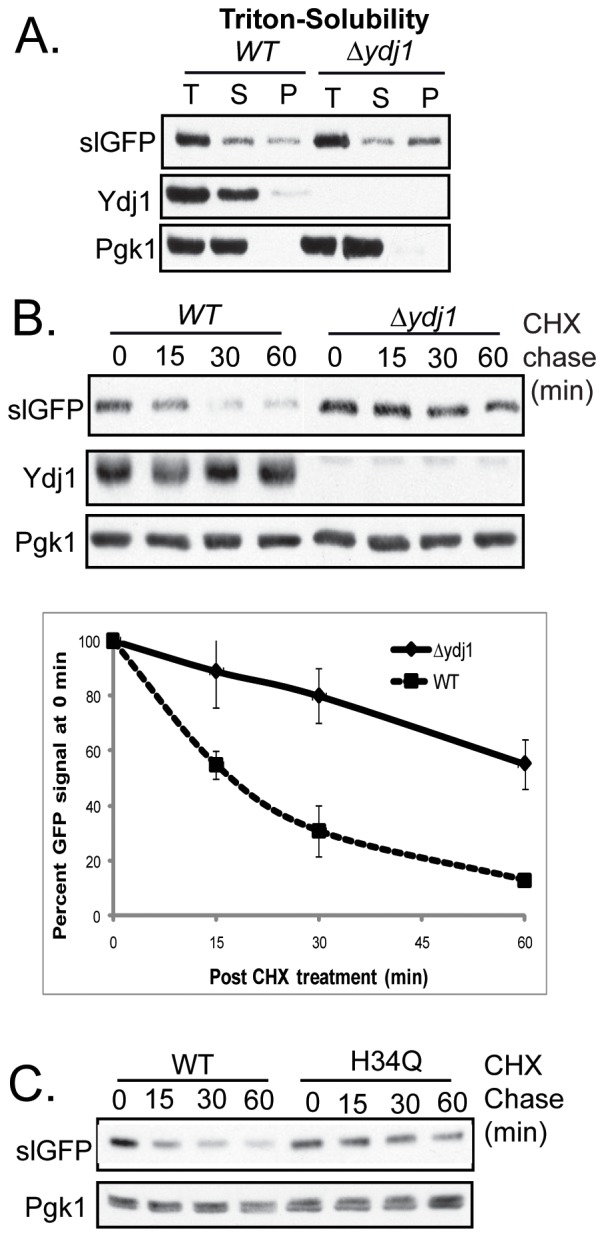
Loss Of Ydj1 Leads SlGFP To Accumulate In A Detergent In-Soluble State. A) Triton X-100 solubility of slGFP after high-speed centrifugation in WT or *Δydj1* (T-total, S-supernatant, P-pellet). B) SlGFP turnover with quantification of slGFP turnover kinetics being shown below the gel, error bars represent ± SEM, N = 3. C) SlGFP turnover in *Δydj1* expressing *YDJ1* or *ydj1*(H34Q) from the authentic *YDJ1* promoter.

Accumulation of Triton X-100 soluble forms of slGFP in puncta in the low Sis1 strain did not appear to result from a general decrease in cytosolic chaperone capacity. This is the case because deletion of the non-essential *YDJ1*, which is several fold more abundant than Sis1 and superior at suppression of protein aggregation [26], resulted in the accumulation of slGFP in Triton X-100-insoluble aggregates (Fig. 5A). Consistent with large pools of slGFP aggregating and becoming insoluble, the degradation of slGFP in *Δydj1* was very inefficient with its half-life being greater than 60 mins (Fig. 5B). Ydj1 cooperated with Hsp70 to maintain slGFP in a soluble state because expression of a form of Ydj1 possessing a mutation in the J-domain could not restore slGFP degradation in a *Δydj1* background (Fig. 5C). We attempted to examine slGFP subcellular localization in *Δydj1*, however in this strain the fluorescent signal of slGFP was not detected (data not shown). Full-length slGFP is detected by western blot in *Δydj1*, so slGFP is translated in this strain, but may not be detected by fluorescence microscopy due to its aggregation prior to proper folding. Thus, in contrast to Sis1 action in degradation of slGFP, Ydj1 may be required to chaperone nascent slGFP.

### PQC E3s Are Required For Sis1 To Accelerate SlGFP Degradation

**Figure 6:**
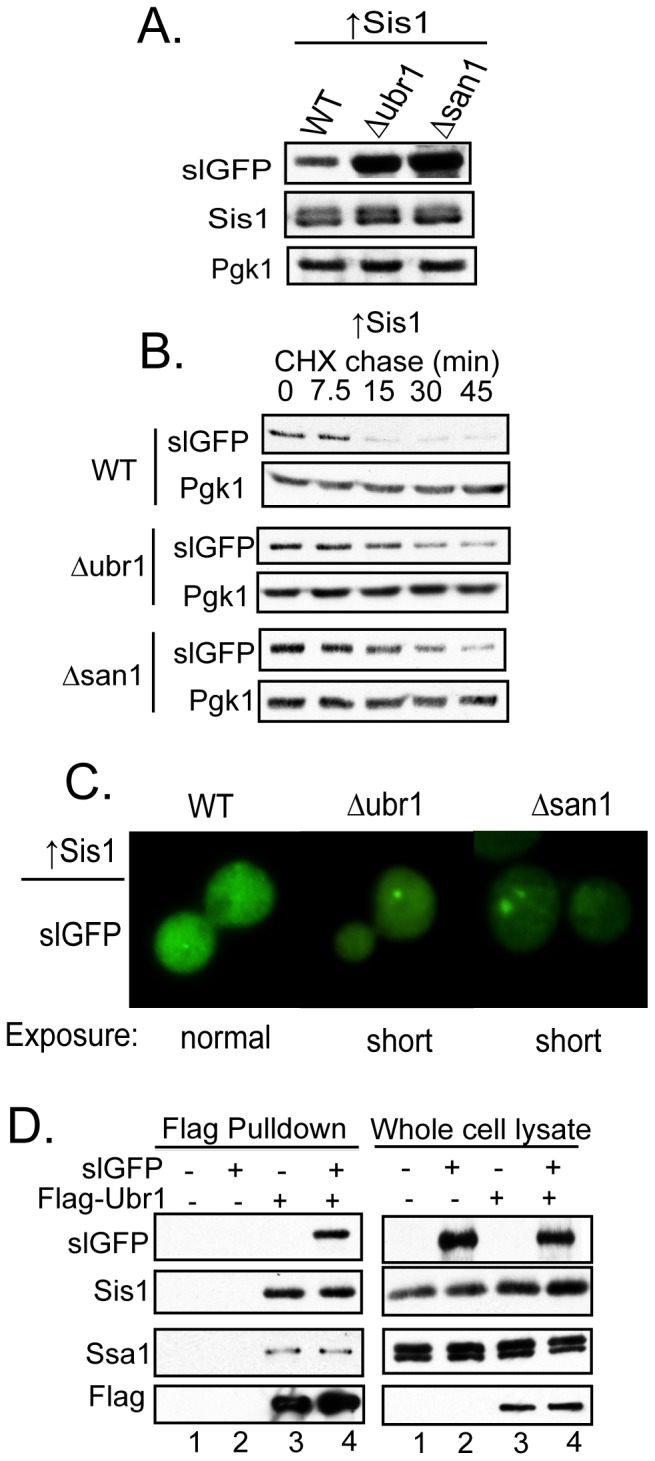
Acceleration Of SlGFP Degradation By Sis1 Requires Ubr1 Or San1. A) SlGFP levels in WT, *Δubr1* or *Δsan1* strains where Sis1 is overexpressed. B) Cycloheximide-chase analysis of slGFP turnover. C) Fluorescence microscopy of slGFP. D) Endogenous Sis1 and slGFP form a complex with Flag-Ubr1. Flag-Ubr1 was isolated from cell extracts using Flag-affinity resin and associated proteins eluted with Flag peptide.

Depletion of Sis1 and deletion of *UBR1* or *SAN1* decreased the rate of slGFP degradation and in each of these instances, slGFP accumulated in Triton X-100-soluble puncta. Therefore, we investigated whether Sis1 function in turnover of short-lived proteins is dependent upon Ubr1 or San1. To test for functional interactions between Sis1 and quality control E3 ligases we first asked if the presence of Ubr1 or San1 is required for Sis1 to accelerate slGFP degradation (Fig. 6). As demonstrated above, Sis1 overexpression accelerates slGFP degradation. However, the deletion of *UBR1* or *SAN1* prevented overexpressed Sis1 from reducing steady-levels of slGFP (Fig. 6A). Furthermore, Sis1 was unable to stimulate rates of slGFP turnover in the absence of either *UBR1* or *SAN1* (Fig. 6B). In addition, the elevation of Sis1 does not block the accumulation of slGFP in puncta that are observed in *Δubr1* and *Δsan1* (Fig. 6C). Sis1 acts on slGFP in a pathway that requires PQC E3 ligases, and Sis1 does not appear to function by simply suppressing slGFP aggregation.

To evaluate the potential for Sis1 to directly interact with PQC E3s we carried out co-precipitation studies with Flag-Ubr1. FLAG-Ubr1 was chosen because San1 is a dynamically unfolded protein and is not amenable for study at the biochemical level in yeast cell extracts [42]. Flag-Ubr1 was expressed and isolated from yeast cell extracts under native conditions via FLAG affinity resin and bound material was then eluted with FLAG peptide. SlGFP, Sis1, and Hsp70 Ssa1 all precipitated with Flag-Ubr1 (Fig. 6D). We were unable to reproducibly detect other QC factors including Ydj1 or Hsp104 in Flag-Ubr1 precipitates (not shown). Sis1 is present in complexes that contain Ubr1 and appears to act at step upstream or coincident with PQC E3's to promote degradation of a terminally misfolded cytosolic protein.

### Overexpressed Sis1 Requires Hsp104 To Accelerate SlGFP Degradation

**Figure 7:**
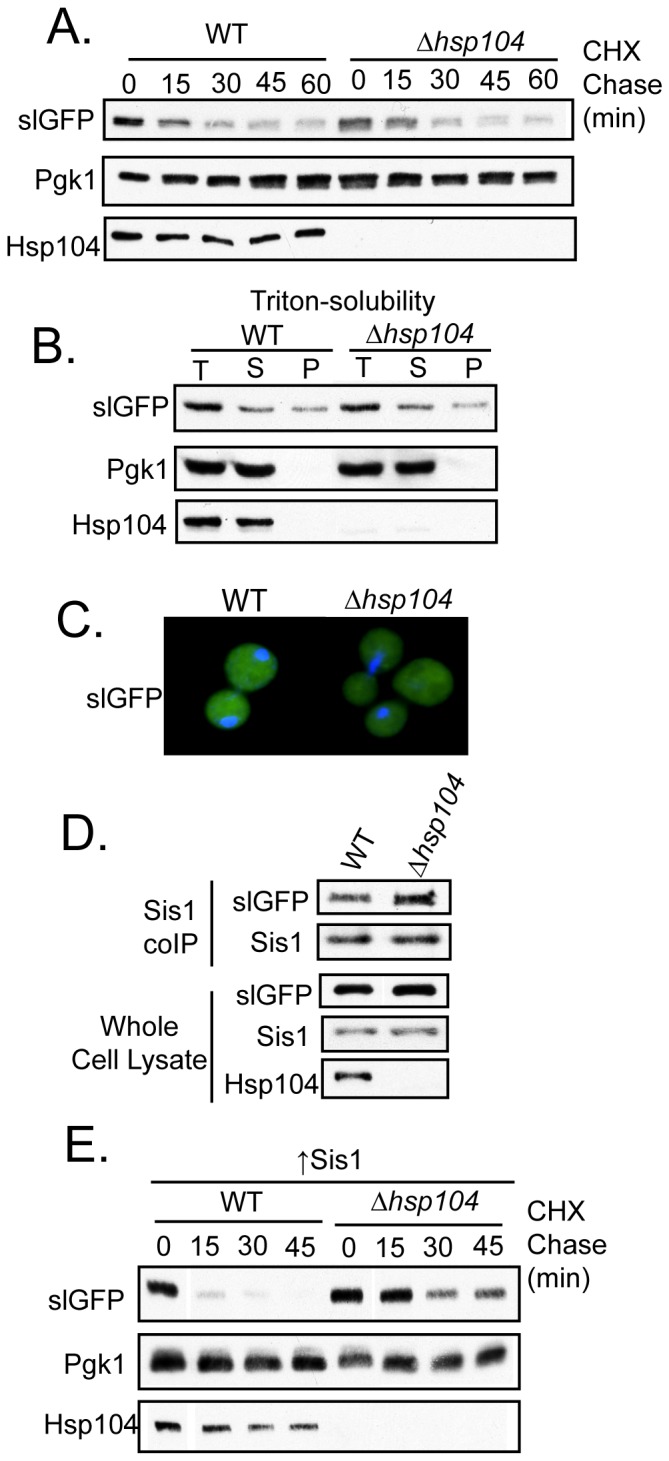
Hsp104 Is Required For Sis1 To Accelerate SlGFP Degradation. A) Cycloheximide-chase analysis of slGFP turnover in WT or *Δhsp104*. B) Triton X-100-solubility of slGFP (T-total, S-supernatant, P-pellet). C) Fluorescence microscopy of slGFP localization D) Co-immunoprecipitation Sis1 and slGFP from a WT or *Δhsp104* strain (upper panel). Total protein levels are shown in the lower panel. E) Cycloheximide-chase analysis of slGFP turnover in a WT or *Δhsp104* strain with overexpressed Sis1.

Our results suggest that perturbation of chaperone function in the QC of slGFP results in accumulation of this misfolded protein in cytosolic puncta that contain detergent soluble proteins. The AAA-type ATPase Hsp104 is present in the JUNQ and peripheral puncta [8], [9] and both Hsp70 Ssa1 and Sis1 help to recruit substrates to Hsp104 [43], [44]. Thus, we asked if Hsp104 plays a role in Sis1 dependent aspects of slGFP degradation. We observed no change in slGFP turnover in *Δhsp104*, or when Hsp104 activity was blocked with Gdn-HCl (Fig. 7A, and data not shown). Furthermore, slGFP remained predominantly diffuse and Triton X-100-soluble in *Δhsp104* (Fig. 7B and 7C). Thus, under normal conditions, Hsp104 does not appear required for slGFP degradation.

Hsp104 does however appear to have an impact on the life and death of slGFP. Levels of immuneprecipitable Sis1:slGFP complexes increase around 1.5 fold in *Δhsp104* (Fig. 7D) and the expression of slGFP induces Hsp104 expression (Fig. 1). We also found that Hsp104 expression, but not that of Hsp70 Ssa1 or Ydj1, is strongly induced upon depletion of Sis1 (Fig. S2). Importantly, Hsp104 is required for overexpressed Sis1 to accelerate slGFP degradation (Fig. 7E). These data suggest that functional interplay between Hsp104 and Sis1 occurs in relation to PQC of slGFP, but in the absence of Hsp104 the cell has the capacity to efficiently degrade slGFP.

### SlGFP That Accumulates In Puncta Is Subsequently Degraded By The Proteasome

Compromise of Sis1 or Ubr1 function extends the slGFP half-life and causes slGFP to accumulate in puncta. Interestingly, in *Δubr1* the half-life of slGFP is increased from approximately 15 min to 90 min (Fig. 2), but in the low Sis1 strain the half-life of slGFP only increases to 30 mins (Fig. 5). These data suggest that in the low Sis1 strain the slGFP observed in puncta is competent for degradation.

**Figure 8:**
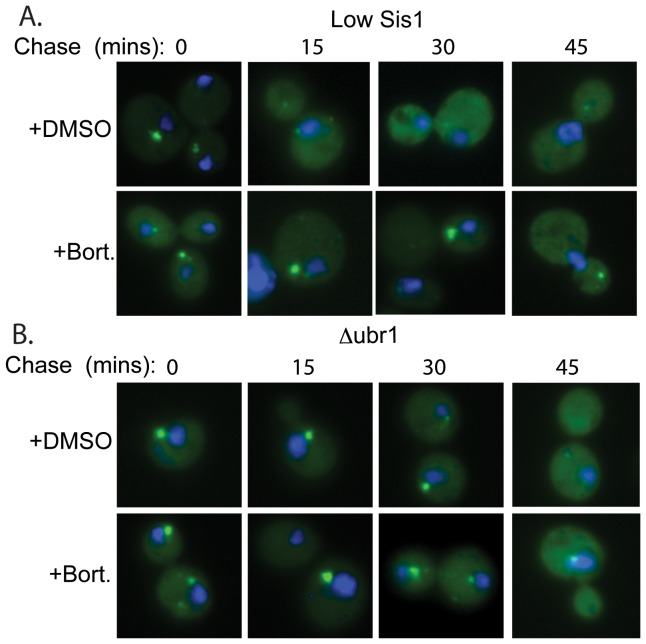
Behavior Of SlGFP Puncta In Cycloheximide Chase Experiments. Time course of slGFP puncta stability in A) low Sis1 or B) *Δubr1*. Strains that express slGFP were grown to mid-log phase and then treated either DMSO or 100 µM bortezomib for 20 min. Cycloheximide was added and cells were incubated for the indicated time and then fixed. slGFP in DAPI stained cells was visualized by fluorescence microscopy.

To address whether puncta localized slGFP that accumulates in low Sis1 or *Δubr1* strains is degradable, we followed the fate of slGFP puncta by fluorescent microscopy in cycloheximide chase studies (Fig. 8). Under conditions of low Sis1, slGFP puncta observed at T = 0 were mostly gone at T = 15 min and this was accompanied by an increase in a diffuse signal at T = 15 and 30 min that started to disappear at 45 min. The rate for disappearance of slGFP puncta in the low Sis1 strain correlated well with half-life of slGFP in this strain. In contrast, slGFP puncta in *Δubr1* were stable for long time periods, with diffuse cytosolic fluorescent patterns being detected at 30 and 45 mins and this fit with the long half-life of slGFP in *Δubr1*. These data imply that inefficient degradation of slGFP leads to its accumulation in puncta as a detergent-soluble species that remains competent for degradation. Puncta-localized slGFP is solubilized and degraded when Sis1 levels are low, but slGFP puncta formed in *Δubr1* are more stable. Ubr1 appears to participate in degradation of non-native proteins that accumulate in cytosolic puncta.

## Discussion

Data presented define a novel a PQC pathway in which the cytosolic Type II Hsp40 Sis1 functions with Hsp70 and PQC E3 ligases (Ubr1 and San1) to mediate proteasomal degradation of a terminally misfolded protein called slGFP. A decrease in the flux of slGFP through the Sis1/Hsp70/PQC E3 pathway results in the accumulation of slGFP in cytosolic puncta. Triton X-100-soluble forms of slGFP that accumulate in puncta are resolubilized and degraded in an Ubr1 and proteasome dependent manner. Thus, Sis1/Hsp70 and PQC E3s cooperate to clear terminally misfolded proteins from the cytosol. Saturation of this system can lead to storage of degradable protein species in puncta that serve as transient holding depots for detergent soluble assemblies of misfolded proteins [8], [9], [48].

When PQC pathways are saturated cells package non-native proteins into several different protein handling centers; the IPOD, JUNQ and peripheral compartment [8], [9], [48]. How non-native proteins are specifically sorted between such compartments and the ultimate fate of these sequestered proteins is under investigation. Ubiquitination is implicated as a targeting signal for protein accumulation in the JUNQ [6], [8], [9], [10], but the fate of proteins that enter the JUNQ is not entirely clear. We find that inhibition of slGFP ubiquitination via deletion of PQC E3 ligases results in its accumulation in a perinuclear location that resembles the JUNQ. Thus, there appear to be multiple modes for routing misfolded proteins to the JUNQ.

Sis1 is found to function in concert with Hsp70 and PQC E3s to mediate degradation of slGFP. In addition, Sis1 appears to cooperate with Hsp104 to accelerate rates of slGFP degradation, but Hsp104 is not required for normal rates of slGFP degradation. Thus, Sis1 seems to function via Hsp104 independent and dependent mechanisms to facilitate slGFP degradation. Sis1 and Hsp70 Ssa1 are present in complexes that contain Ubr1, and Hsp70 is known to function in substrate selection by PQC E3s [2], [3]. Thus, Sis1 and Hsp70 Ssa1 are likely to function independent of Hsp104 in slGFP degradation via assisting in the selection of non-native proteins for ubiquitination by Ubr1. Sis1 and Hsp70 may also play a similar role in San1 function. However, we the dynamic instability of San1 prevented us from exploring this possibility.

To accelerate the rate of slGFP degradation Sis1 may function with Hsp104 in a manner similar to Sis1/Hsp104 function in prion propagation [9], [43], [45], [46], [47]. In this process Sis1 and Hsp70 recruit oligomeric prions to Hsp104 for disaggregation [9], [43], [47]. The aggregation state of slGFP is sensitive to subtle perturbations in PQC, so it is likely that pools of slGFP exist in equilibrium between monomeric and small aggregated species that are not observed by fluorescence microscopy. Sis1 may therefore accelerate slGFP degradation rates via cooperation with Hsp104 to increase the pool size of monomeric slGFP, which would in turn increase the rate of slGFP ubiquitination by PQC E3 ligases.

The half-life of puncta that contain slGFP in low Sis1 or *Δubr1* strains is different. SlGFP present in the puncta of the low Sis1 strain is resolubilized and degraded in a proteasome-dependent manner. Yet, in *Δubr1* the puncta that contain slGFP are relatively stable and slGFP has a half-life of greater than 90 mins instead of 15 min. These data suggest that slGFP degradation intermediates accumulate in cytosolic puncta when the capacity of Sis1 and Hsp70 to handle non-native proteins is saturated. In addition, it appears that Ubr1 is required for degradation of misfolded proteins that are liberated from the puncta. Further studies are now required to understand the functional interplay between PQC factors that degrade non-native proteins and those that package non-native proteins for reversible sequestration into protein storage depots. A mechanistic understanding of these cellular processes will help define approaches to suppress the proteotoxicity associated with protein conformational disease.

## Supporting Information

Figure S1 **The Domain Structure Of SlGFP.** A) Sequence and predicted secondary structure of the N-terminal 120aa of slGFP; C-coiled H-helical S-β-strand. B) Turnover of slGFP wild type and mutant lacking its nuclear localization sequence (NLS) and nuclear export sequence (NES). C) Fluorescence microscopy of slGFP and NLS/NES mutant.
(TIF)

Figure S2 **Depletion Of Sis1 To Undetectable Levels Delays SlGFP Turnover.** A yeast strain expressing slGFP as described in Figure 4 (low Sis1) was treated with or without doxycycline to deplete Sis1. The turnover of SlGFP was analyzed in a cycloheximide chase time course. Changes in chaperone levels were compared in the absence or presence of doxycycline.
(TIF)

Table S1 **Genotypes Of Yeast Strains Used In Study Of The SlGFP Degradation.**
(DOCX)

Table S2 **Plasmids Utilized In The Study Of SlGFP Degradation.**
(DOCX)
