# Supplementary figures and images for: The Type II Hsp40 Sis1 Cooperates with Hsp70 and the E3 Ligase Ubr1 to Promote Degradation of Terminally Misfolded Cytosolic Protein
Source: PLoS One. 2013 Jan 16;8(1):e52099. doi: 10.1371/journal.pone.0052099 (PMC3547041; doi:10.1371/journal.pone.0052099)

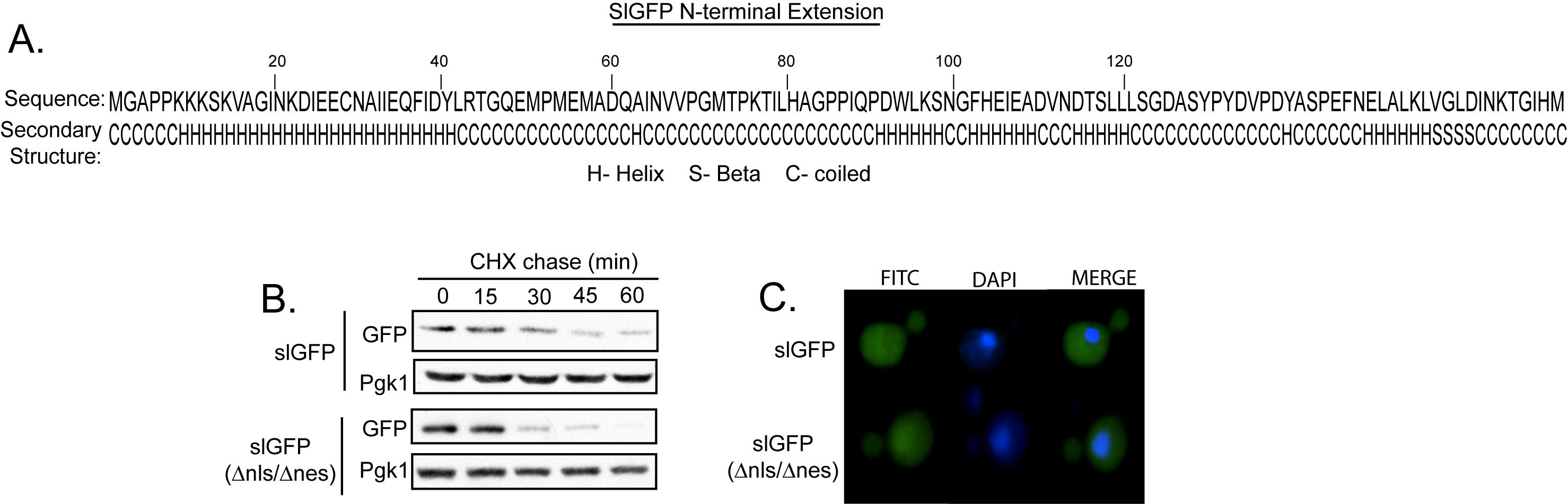

Supplement: Figure S1 — The Domain Structure Of SlGFP. A) Sequence and predicted secondary structure of the N-terminal 120aa of slGFP; C-coiled H-helical S-β-strand. B) Turnover of slGFP wild type and mutant lacking its nuclear localization sequence (NLS) and nuclear export sequence (NES). C) Fluorescence microscopy of slGFP and NLS/NES mutant. (TIF) [file pone.0052099.s001.tif]

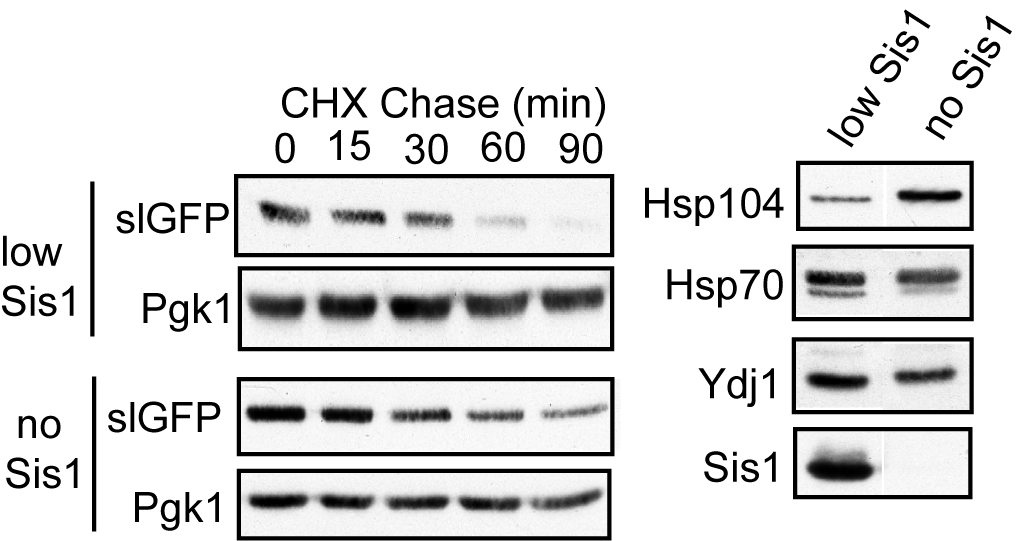

Supplement: Figure S2 — Depletion Of Sis1 To Undetectable Levels Delays SlGFP Turnover. A yeast strain expressing slGFP as described in Figure 4 (low Sis1) was treated with or without doxycycline to deplete Sis1. The turnover of SlGFP was analyzed in a cycloheximide chase time course. Changes in chaperone levels were compared in the absence or presence of doxycycline. (TIF) [file pone.0052099.s002.tif]
