# Supplementary material for: The Type II Hsp40 Sis1 Cooperates with Hsp70 and the E3 Ligase Ubr1 to Promote Degradation of Terminally Misfolded Cytosolic Protein
Source: PLoS One. 2013 Jan 16;8(1):e52099. doi: 10.1371/journal.pone.0052099 (PMC3547041; doi:10.1371/journal.pone.0052099)
Supplement: Table S1 — Genotypes Of Yeast Strains Used In Study Of The SlGFP Degradation. (DOCX) [file pone.0052099.s003.docx]

Table S1

| **Yeast Strain** | **Genotype** | **Source** |
| --- | --- | --- |
| BY4741 WT | *mat a* *∆ura3, ∆leu2, ∆his3, ∆met15* | Open Biosystems |
| *∆ubr1* | BY4741 *∆ubr1: KanMX* | This study |
| *∆san1* | BY4741 *∆san1:KanMX* | This study |
| *∆doa10* | BY4741 *∆doa10: KanMX* | This study |
| BY4742 WT | *mat α* *∆ura3, ∆leu2, ∆his3, ∆lys2* | Open Biosystems |
| *∆pdr5* | BY4742 *∆pdr5:KanMX* | Open Biosystems |
| *∆hsp104* | BY4742 *∆hsp104:KanMX* | Open Biosystems |
| Sis1(tetR) | pSIS1::*kanR-tet07-TATA URA3*::*CMV*-tTA MATa *his3-1* *leu2-0 met15-0* | Open Biosystems |
