# Supplementary material for: The Type II Hsp40 Sis1 Cooperates with Hsp70 and the E3 Ligase Ubr1 to Promote Degradation of Terminally Misfolded Cytosolic Protein
Source: PLoS One. 2013 Jan 16;8(1):e52099. doi: 10.1371/journal.pone.0052099 (PMC3547041; doi:10.1371/journal.pone.0052099)
Supplement: Table S2 — Plasmids Utilized In The Study Of SlGFP Degradation. (DOCX) [file pone.0052099.s004.docx]

Table S2

| Plasmid name | Expression construct | Source |
| --- | --- | --- |
| pKW430 (slGFP) | pRS426 *ADH1* slGFP | K. Weis |
| pKW430 (slGFP ∆nls∆nes) | pRS426 *ADH1* slGFP (∆nls∆nes) | This study |
| pRS425 slGFP | pRS425 *ADH1* slGFP | This study |
| pCUPslGFP | pRS416 *CUP1* NLS-NES-GFP(2X) | This study |
| pGPDYDJ1 | pRS315 *GPD1 YDJ1* | Douglas *et al* 2009 |
| pGPDSIS1 | pRS315 *GPD1 SIS1* | Douglas *et al* 2009 |
| pGPDSIS1H34Q | pRS315 *GPD1 SIS1(H34Q)* | This study |
| pESC-LEU-GFP-VHL | pGAL GFP-VHL | Kaganovich *et al* 2009  Addgene # 21053 |
| pFlagUBR1 | pRS425 *ADH1* Flag-Ubr1 | A. Varshavsky |
| pGALRnq1mRFP | pRS416 *GAL1 RNQ1-mRFP* | Douglas *et al* 2008 |
